# Coupled DEM-FDM analysis of open-ended pile installation and lateral loading in chalk

**DOI:** 10.1007/s11440-025-02905-3

**Published:** 2025-12-22

**Authors:** Jinhui Zheng, Matteo O. Ciantia, Jonathan A. Knappett

**Affiliations:** 1 School of Science and Engineering, University of Dundee, Nethergate, Dundee, DD1 4HN UK; 2 Department of Engineering Science, University of Oxford, Parks Road, OX1 3PJ, Oxford, UK; 3 Department of Earth and Environmental Sciences, University of Milano-Bicocca, Piazza della Scienza, 4, 20126, Milan, Italy

**Keywords:** Chalk, Coupled DEM-FDM modelling, Installation effects, Rock–pile interaction

## Abstract

The in-service capacity of monopiles for offshore wind turbines founded in chalk is a function of the state of the rock around the pile. The installation of a displacement pile affects the fabric of the surrounding rock (‘installation effects’). In this paper, a coupled DEM (discrete element method)-FDM (finite differential method) 3D model is used to investigate the installation effects of open-ended displacement piles and their influence on subsequent lateral behaviour in soft rocks. Results indicate that the axial resistance of installed piles in chalk is primarily provided by external shaft and base resistances. In addition, rock material inside the pile cavity experiences a significant dilation behaviour, suggesting traditional indices (incremental filling ratio (IFR) and plug length ratio (PLR)) are unsuitable for evaluating pile installation mode. Considering the distribution of the principal stress, the axial load transfer mechanism for the pile is proposed from a microscale perspective. Subsequent lateral loading simulation reveals that changes in the rock state around the pile due to installation strongly influence lateral stiffness. Finally, macro- and micro-scale analyses confirm that the dominant contributors to lateral resistance are the pile tip region on the passive side and the near-surface region on the active side of the pile.

## Introduction

In recent years, offshore wind energy has emerged as the preferred form of green energy for north-European countries due to their abundant wind resources. In this scope, most existing offshore wind farms are situated in areas with water depths less than 30 m and employ monopile foundations due to their high bearing capacity [1, 63, 74]. During the installation of open-ended piles, the material entering the pile can induce significant friction against the internal walls of the pile, forming a plug that prevents additional material from entering the pile. This is typically referred to as a “plugged” condition. Pile installation can also be unplugged (i.e. coring) and partially plugged. Different plug states have a significant negative effect on pile drivability and load capacity [34, 48].

European wind farms are often located on soft rocks, such as chalk [7, 8, 11]. The primary factors affecting pile drivability and axial bearing capacity in low- to medium-density chalk include end resistance and external and internal shaft resistances. In soil, the relative magnitudes of internal and external shaft resistances are generally closely related to the pile diameter-to-wall thickness ratio and soil conditions (e.g. relative density in sands) [18, 19, 32–34, 41]. In highly porous, soft rocks, however, the installation involves bond breakage and particle crushing, sometime leading to pile free-fall self-weight ‘runs’ as collapsible microstructure, which further complicates the relationship between internal and external shaft friction [29].

To establish a design suitable for monopiles in chalk strata, the joint industry project ALPACA (axial-lateral pile analysis for chalk applying multi-scale field and laboratory testing) conducted systematic research combining advanced field and laboratory tests [2, 7, 8, 38, 62]. The study revealed that the pile driving process remodels the chalk structure, forming fine-grained annuli on both the internal and external sides (chalk putty) [46]. The development of effective stress paths at the pile–annulus interface exhibits similarities to analogous tests in sands, with failure modes following the Coulomb failure criterion and demonstrating constrained interface dilatancy. Consequently, based on the ICP-05 design method for sand [28], a newly proposed version, ICP-18, was proposed for chalk formations [30]. The ICP-18 method assumes negligible internal shaft friction for offshore monopiles with a large diameter to wall thickness ratio, and this assumption has been validated through signal-matching inversion techniques based on Fibre Bragg Grating (FBG) measurements [9]. This approach effectively addresses the overly conservative estimation of shaft friction (20 kPa) for low- to medium-density chalk in traditional design recommendations [40], providing a more rational basis for the drivability and axial capacity design of open-ended piles in low- to medium-density chalk. However, ICP-18 is primarily based on a limited number of field tests, and the understanding of penetration mechanisms or installation effects caused by pile–chalk interaction remains insufficient. Therefore, further investigation through numerical modelling is warranted.

To accurately evaluate installation effects, continuum-based numerical approaches, such as PFEM (particle finite element method), MPM (material point method), CEL (coupled Eulerian–Lagrangian method) and ALE (Arbitrary Lagrangian–Eulerian), have recently been employed to model pile installation in soils and rocks [21, 45, 54, 59, 60, 64, 69]. The predictive capability of continuum-based modelling is inherently constrained by its constitutive framework. Such models generally cannot resolve key micromechanical processes, including particle rearrangement and force chain evolution, both of which are critical for elucidating the underlying mechanism of open-ended pile installation. The axial load transfer mechanism of OE piles in sand is based on arching [51, 52] and has been shown to be captured using discrete numerical modelling such as DEM [10]. Although pile installation in granular materials has been investigated by many scholars [4, 14, 20, 35–37], only Zhang and Fatahi [70] used DEM to analyse the installation effect of OE piles in cemented soft rocks. However, their simulations, which required a runtime of more than two months, could not simulate large-scale boundary value problems (BVPs) efficiently.

Once in service a monopiles’ lateral response is influenced by the superstructure’s natural frequency, which depends on the foundation’s stiffness [21]. This stiffness is closely tied to the initial state of the rock surrounding the pile shaft. The installation process alters the rock’s microstructure (e.g. by changing porosity and bond degradation), often resulting in a stiffer response and increased lateral capacity. Despite this, most reported studies simulate laterally loaded piles using a wished-in-place (WIP) approach disregarding installation effects [5, 16, 17, 61].

This paper aims to efficiently simulate installation effects of monopile in soft rocks and their influences on subsequent lateral behaviour. Two model piles with different section sizes and aspect ratios are considered, along with a further case applying WIP installation to investigate the effect of installation method on lateral response. To properly simulate the stress state of the pile and its deformation (e.g. Poisson straining [30]), FDM elements are used to simulate the pile structure, while a coupled DEM-FDM approach is adopted to enhance the computational efficiency [65, 72]. To accurately replicate the softening behaviour of cemented bonds, a recently developed damage bonded contact model is implemented within the DEM zone [73].

## Novel damage bonded model

The bond damage contact model adopted in this study has been proposed and validated in Zheng and Ciantia [73], where a detailed description can be found. Here, only a brief summary of its key features is provided for completeness. The model is implemented as a user-defined contact model in PFC3D [26].

### Overview of contact law

The model describes the evolution of bonded and unbonded contact forces between particles. The bonded interaction comprises normal and shear forces, as well as a bending moment, while the unbonded interaction is described by a conventional linear contact model. Bond deformation includes both elastic and plastic parts, and the bond damage is characterised by a scalar parameter ${D}_{d}$, which evolves with plastic contact deformation to capture the ductile softening behaviour typical of porous soft rocks such as chalk. By definition, ${D}_{d}$ = 0 corresponds to an intact bond, whereas ${D}_{d}$ = 1 indicates complete bond breakage. The full constitutive formulation, yield surface definition, and numerical integration scheme are provided in Zheng and Ciantia [73].

**Fig. 1:**
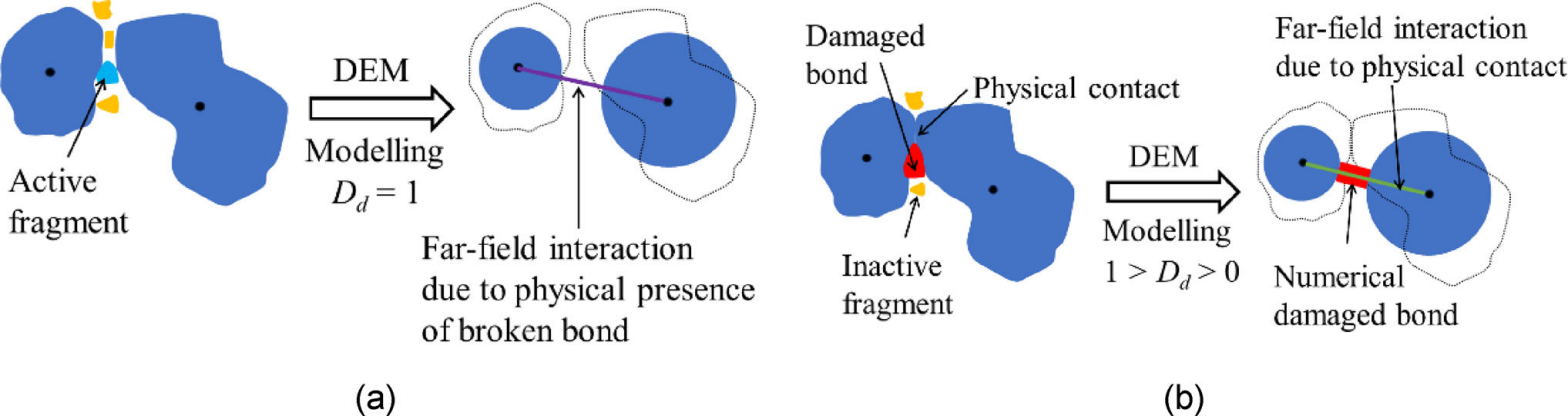
Schematic diagram of the far-field interaction

A key feature of this model is the introduction of far-field interactions, which control the activation of unbonded forces. Chemically formed cemented bonds break into several fragments, with some fragments remaining between non-contacting grains hence allowing to transfer some load [13]. This mechanism is incorporated via far-field interactions, as illustrated in Fig. 1a. Furthermore, when bonds are damaged but not yet fully broken (0 < ${D}_{d}$ < 1), spherical particles in DEM may appear physically separated, while irregular-shaped grains in reality would still have established physical contact (Fig. 1b). Incorporating far-field interactions allows the model to accurately represent this situation. The combination of the damage variable and far-field interactions enables the model to capture the transition from brittle to ductile behaviour in soft rocks under complex stress conditions.

### Model calibration

Model parameters for chalk are calibrated against the results of isotropic and triaxial compression tests from Liu et al. [39], the procedures for which are described in Zheng and Ciantia [73]. The calibrated chalk, a typical porous, soft rock distributed in the North Sea, has a porosity of 0.45 and UCS of approximately 2.8 MPa.

**Fig. 2:**
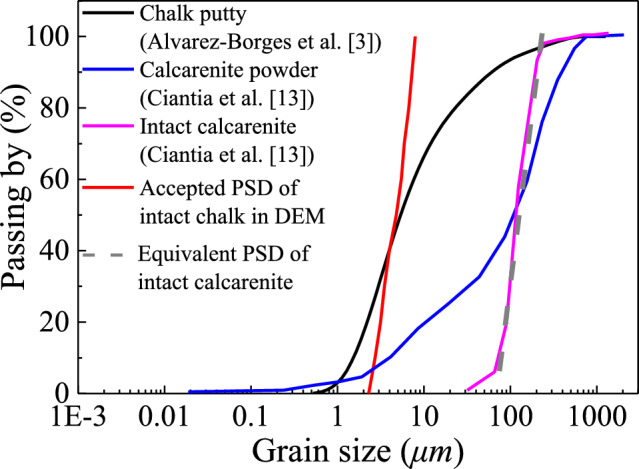
Particle size distribution of simulated material

Figure 2 presents the particle size distribution (PSD) of this soft rock and the PSD used in DEM. Currently, only the PSD of destructured chalk (putty) is available. Given that chalk and calcarenite are both porous carbonate soft rocks with comparable structure and mechanical behaviour, the PSD of intact calcarenite is used as a proxy for intact chalk. To meet DEM numerical constraints on particle-size ratios, the intact calcarenite PSD is first equivalently simplified. Comparison shows that intact and destructured calcarenite have similar d_50_. Based on this, the engineering assumption is adopted that intact and destructured chalk also shares a similar d_50_. Under this assumption, the “equivalently simplified intact calcarenite PSD” is shifted along the size axis to match the d_50_ of chalk putty, yielding a simplified PSD for intact chalk. Please note that the PSDs of destructured chalk and destructured calcarenite are not identical (the latter is broader), so this shift is a reasonable engineering approximation. Numerous DEM studies have shown that, even with simplified or randomly generated PSDs, simulations can reproduce experimental trends at both macro- and micro-scales [37]. Therefore, the proposed treatment is acceptable and practical for engineering analysis.

**Fig. 3:**
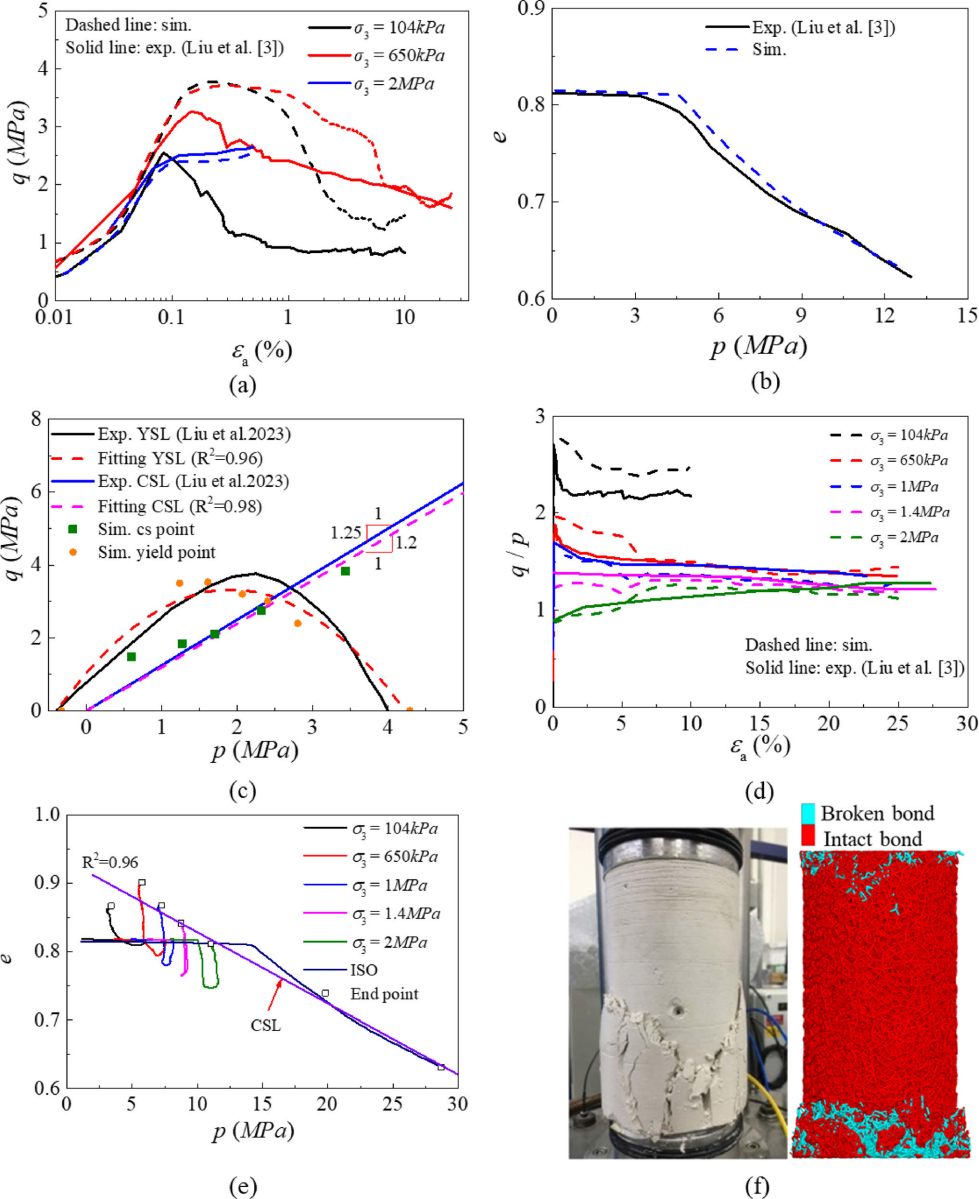
Calibrated results: **a** triaxial and **b** isotropic tests; **c** YSL and CSL; **d** *q*/*p* vs. ${\varepsilon }_{a}$; **e** *e* vs. *p*; **f** failure mode (104 kPa) from Pedone et al. [53]

The simulated chalk sample consists of approximately 24,000 particles and has a height of 76 mm, a diameter of 38 mm and initial porosity of 0.45. The final calibrated parameters and the adopted PSD (particle upscaling ratio = 15) are summarised in Table 1. As shown in Fig. 3a–d, overall a good agreement is observed in both triaxial and isotropic compression tests (note YSL = yield surface line; CSL = critical state line). As the experimental yield envelope is capped, the yield strength of the 104 kPa and 650 kPa confinement pressure tests is quite similar. However, in the experimental results, the post-peak response under low confinement is affected by strain localisation (Fig. 3f), which induces a non-homogeneous deformation within the sample. This explains why the results of test 104 kPa do not agree with the critical state (CS) response observed in the other simulations, which did not exhibit any localised failure patterns. Under low confining pressure, rock failure is dominated by tension-induced cracking, which triggers pronounced stress softening and strain localisation, ultimately forming narrow localised failure bands. In contrast, at high confining pressure, crack opening is suppressed and the failure mechanism shifts towards shear sliding and plastic spreading, leading to more uniform deformation and the absence of localised failure.

**Table 1:** Calibrated model parameters and employed PSD for intact chalk

Parameter	Value	Parameter	Value
*Unbonded* *part*			
${E}_{\mathrm{mod}}$	0.63 GPa	${\kappa }^{*}({\overline{\kappa }}^{*})$	5
${\overline{E} }_{\mathrm{mod}}$	5.5 GPa	$\mu$	0.2
*Bonded* *part*			
${\sigma }_{c}$	6 MPa	${u}_{c}^{n}$(${u}_{c}^{s}$)	0.03d_00_
${\sigma }_{t}$	1.5 MPa	${\theta }_{c}^{b}$	0.01
$C$	1.5 MPa	${g}_{a}$	0.13d_00_
*PSD* *information*			
d_00_	0.983 mm	d_10_	1.167 mm
d_20_	1.350 mm	d_30_	1.493 mm
d_40_	1.618 mm	d_50_	1.747 mm
d_60_	1.871 mm	d_70_	2.024 mm
d_80_	2.222 mm	d_90_	2.473 mm
d_100_	2.708 mm	–	–

$${u}_{c}^{n}$$=0.23d_00_ for compression

## Coupled FDM-DEM modelling of pile installation

### Model information

The installation process of two small scale piles (tested at 1 − g) is investigated in this section. The model pile experiments that are simulated are reported in Riccio [56], Riccio and Ciantia [57] and Alvarez-Borges et al. [3]. The pile geometries and pile–chalk interface contact parameters are listed in Table 2. The chalk-steel friction angle is about 30° in laboratory tests [53], while a smaller chalk-steel friction coefficient ${\mu }_{\mathrm{pile}-\mathrm{chalk}}$ is adopted in Table 2 since model piles are fabricated from polymethyl methacrylate (PMMA, [75]). Additionally, ${\kappa }^{*}=8$ is determined from Wen et al. [66].

**Table 2:** Pile geometry and pile–chalk contact information

	Pile A Alvarez-Borges et al. [3]	Pile B Riccio [56]
${D}_{\mathrm{pile}}$	8 mm	18.95 mm
${t}_{w}$	1 mm	0.95 mm
${t}_{w}/{\mathrm{d}}_{50}$	3	2
${L}_{p}$	32 mm	36.5 mm
${L}_{\mathrm{pile}}$	60 mm	228 mm
${E}_{\mathrm{mod}}$	1 GPa	1 GPa
${\kappa }^{*}$	8	8
${\mu }_{\mathrm{pile}-\mathrm{chalk}}$	0.3	0.3

Deformation between the discrete and continuous regions is transmitted through a coupling wall attached to the FDM elements at the interface [68]. The coupling strategy operates through the following mechanism: Contact forces between particles and the coupling wall are first converted into equivalent nodal forces applied to FDM element nodes; the deformation response of FDM elements is then computed based on the constitutive model, followed by updating the spatial position of the segments of the coupling wall according to the element deformation; finally, contact forces between particles and the coupling wall are recalculated through the updated geometric relationship at the coupling wall. Further technical details can be found in Zheng et al. [72].

**Fig. 4:**
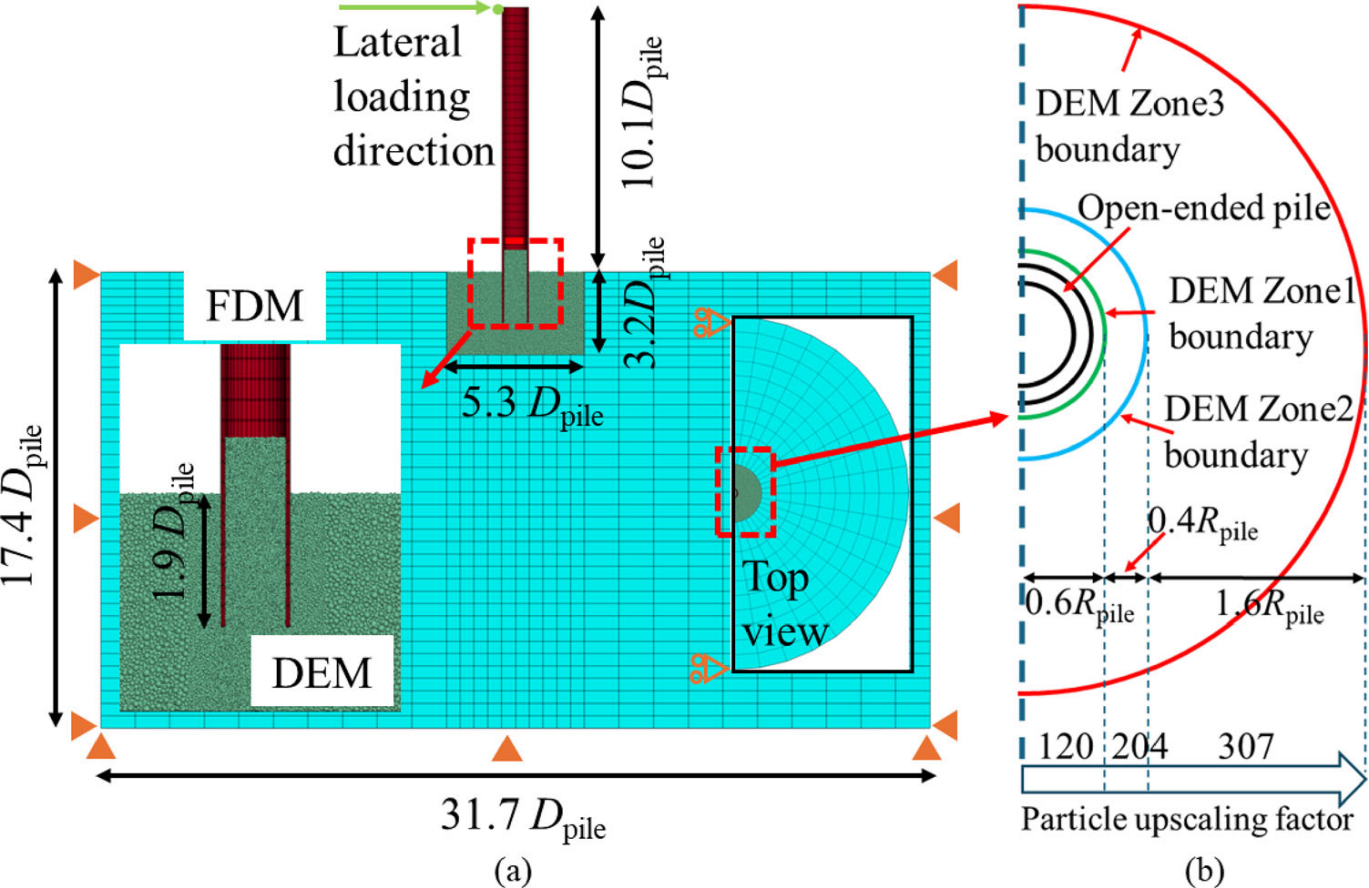
**a** model layout and **b** particle upscaling ratio in DEM region of Pile B

As irreversible damage is focussed close to the pile, the constitutive model employed in the FDM region (see Fig. 4) is isotropic elastic, characterised by the elastic parameters of the intact rock (Young’s modulus and Poisson’s ratio of 3 GPa and 0.2, respectively). The geometries of the DEM and combined regions are given in Table 3.

**Table 3:** Size information of coupled model

	Pile A Alvarez-Borges et al. [3]		Pile B Riccio [56]	
	Diameter (mm)	Height (mm)	Diameter (mm)	Height (mm)
DEM region	40	60	100	60
Whole coupled model	100	135	600	330

**Fig. 5:**
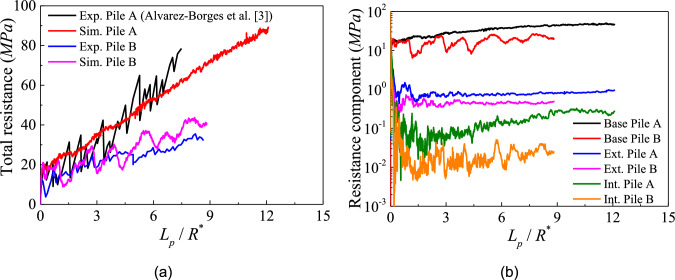
Comparison of installation resistance vs. installed depth of **a** total resistance and **b** resistance components

Initial conditions are reproduced using the periodic cell replication method proposed by Ciantia et al. [12] and Zheng et al. [72]. Initial stress conditions consider a lateral earth pressure coefficient ${K}_{0}$ of 0.6 [39, 62]. Particle scaling was used to increase model efficiency without affecting the response of the contact model [73]. Particle refinement is used to obtain an adequate ${t}_{w}/{\mathrm{d}}_{50}$ ratio at the interface with the pile, where the upscaling ratios in each zone are indicated in Fig. 5b [44]. Although the ${t}_{w}/{\mathrm{d}}_{50}$ ratio in these simulations is smaller than field situations, it is still large enough to avoid particle size effects [10].

### Installation and lateral loading programme

Two piles are jacked to their prescribed depths at jacking velocity ${v}_{\mathrm{pile}}$ determined according to Eq. 1 to ensure quasi-static conditions (${I}_{\mathrm{inner}}<0.01$) [27]:

1 $${v}_{\mathrm{pile}}=3{D}_{\mathrm{pile}}\frac{{I}_{\mathrm{iner}}}{{\mathrm{d}}_{50}}\sqrt{\frac{{\sigma }_{3}}{\rho }}$$

where ${\sigma }_{3}$ is the confinement of the sample; ${I}_{\mathrm{iner}}$ is the inertial number of the model (0.01 used in this study), and $\rho$ is the rock density.

For subsequent lateral loading, a constant velocity is only applied on one edge node at the top of Pile B. The loading was quasistatic, and in this case this is achieved by loading at a rate such that the acting contact force and the unbalanced force of the force applied were equal [31]. The maximum loading rate to achieve this is 0.4 m/s.

## Installation results

### Macro-response

#### Penetration resistance

Figure 5 presents the evolution of installation resistance with depth of the two model piles alongside experimental data. Pile A is from Alvarez-Borges [3], while pile B is from Riccio [56]. The installed depth is normalised by each pile’s equivalent radius ${R}^{*}$ defined as $\sqrt{{R}_{o}^{2}-{R}_{i}^{2}}$, with total and base resistances normalised by the effective area ($\pi {{R}^{*}}^{2}$), where ${R}_{o}$ and ${R}_{i}$ are the external and internal radii of the pile section, respectively. External and internal integrated shaft forces are normalised by their external and internal surface areas, respectively, to obtain shear resistance. A good agreement between simulated results and experimental data is captured in Fig. 5a. In addition, the relatively lower ${t}_{w}/{d}_{50}$ value in Pile B causes the noisier response in Fig. 5b.

Notably, Pile A exhibits higher base resistance and external and internal shaft resistances than Pile B (compared at the same depth normalised by corresponding ${R}^{*}$). This substantial difference can be explained by the ratio of pile diameter to pile wall thickness $D/{t}_{w}$. These ratios are 8 and 20 for Pile A and B, respectively. A smaller $D/{t}_{w}$ ratio corresponds to a larger base area, which enhances the confinement effect on the underlying rock beneath the pile wall, thereby increasing the base resistance. Moreover, this reduced $D/{t}_{w}$ ratio promotes the development of higher internal shaft resistance due to the improved lateral constraint.

#### Stress distributions

**Fig. 6:**
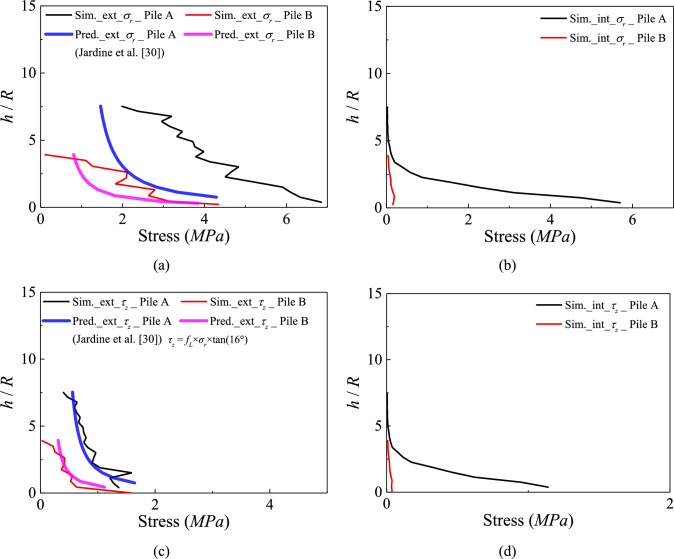
**a** and **b** external and internal radial stresses; **c** and **d** external and internal shear stresses distribution along pile shaft

Figure 6 presents the distributions of radial and shaft stresses acting on the internal and external portions of the pile shaft at the end of installation. For both model piles, the external stresses (both components) are greater than the internal ones. The internal radial and shaft resistances in Pile A (thicker one, *D* / *t*_*w*_ = 8) are 24% of the external resistances, which is higher than what assumed in field tests in the same chalk [8]. In contrast, the internal radial and shaft resistances of Pile B (thinner one, *D* / *t*_*w*_ = 20) are only 4% of the external resistances. Figure 6 also compares the results with the prediction model by Jardine et al. [30]. The cone tip resistance ${q}_{t}$ measured by in situ CPT (cone penetration test) ranges from 10 to 20 MPa [7]. A CPT simulation was also conducted in the same numerical sample, and the simulated ${q}_{t}$ is 15.8 MPa. For simplicity, ${q}_{t}$ was uniformly taken as 15.8 MPa in the Jardine et al. [30]’s prediction. Furthermore, the value of ${f}_{L}$=1.33 is applied in accordance with Jardine et al. [30]. As shown in Fig. 6a, the predicted radial effective stress around Pile A is significantly lower than the simulated values, which is likely attributed to boundary effects caused by the relatively small size of Pile A’s coupled model [49]. The predicted shear stresses in Fig. 6c show good agreement with the simulated results, achieved by using the interface friction angle consistent with the model piles considered in the present simulations (16°, see Table 2).

#### Plugging

**Fig. 7:**
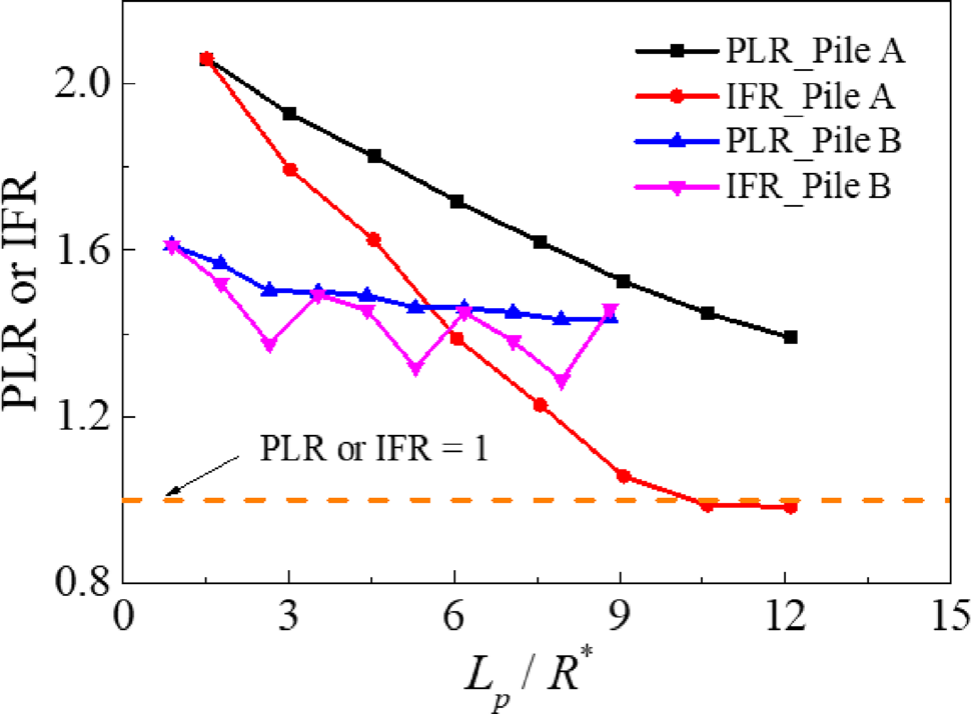
Evolution of PLR and IFR

In Fig. 7, plug length ratio (PLR) and incremental filling ratio (IFR), defined as the ratio of plug length to penetration and its incremental formulation, respectively, are plotted. According to Fig. 7, both Piles A (thicker one,* D* / *t*_*w*_ = 8) and B (thinner one, *D* / *t*_*w*_ = 20) are in an unplugged state (PLR and IFR > 1) [22, 50, 71], although the values are numerically different as these factors vary with the material dilatant behaviour, especially if particle crushing is considered.

For Pile A, the internal shaft resistance increased rapidly during installation, which considerably restrained the growth of the plug height, resulting in a sharp decline in the PLR and IFR with penetration. In contrast, for Pile B, the internal shaft resistance developed gradually. The low internal resistance was insufficient to prevent the influx of external particles, and thus, the incremental rate of change of the plug height remained relatively unchanged throughout the installation process.

### Micro-mechanical analysis

#### Porosity

**Fig. 8:**
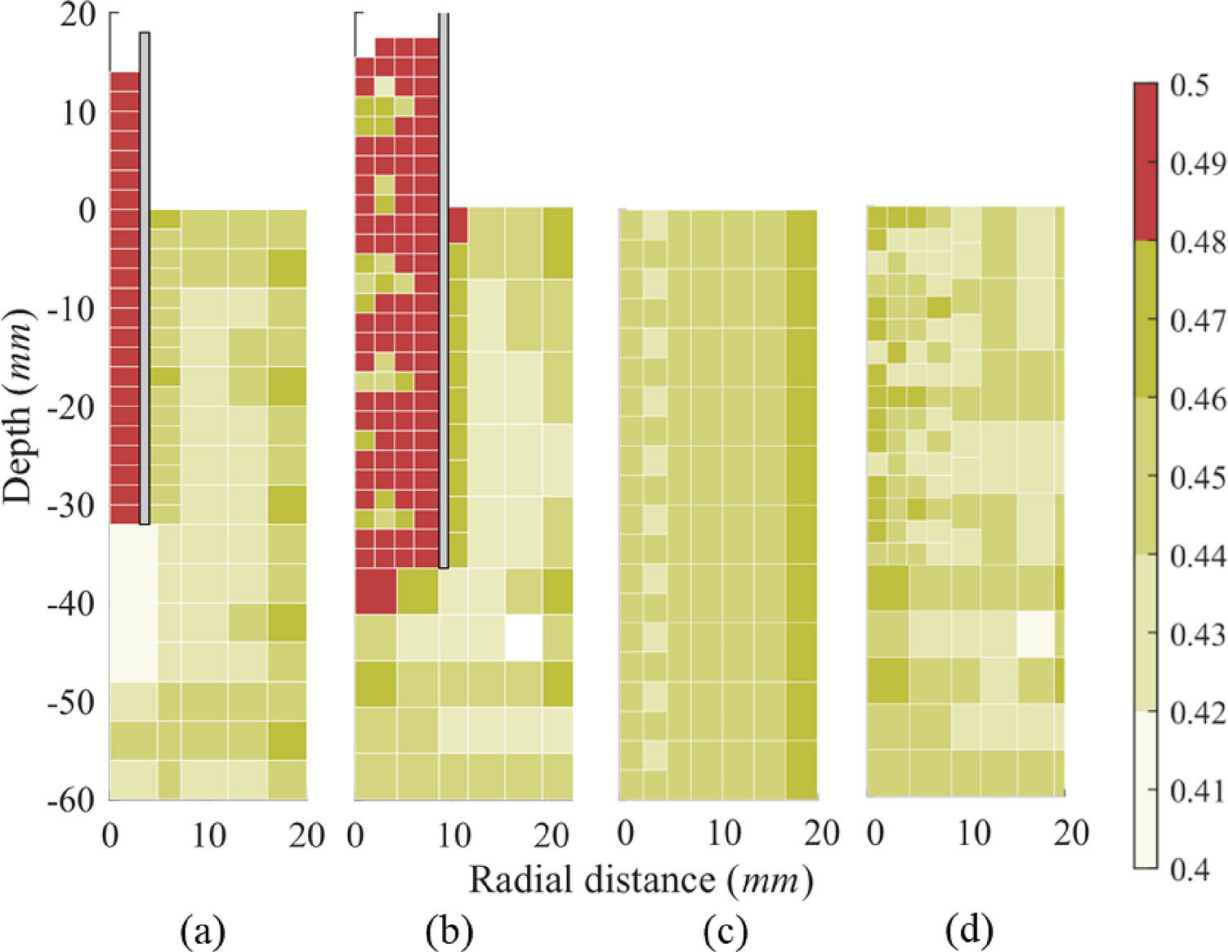
Porosity distribution in the DEM region: **a** Pile A and **b** Pile B after installation; **c** Pile A and **d** Pile B before installation

Figure 8 shows the porosity distribution in the DEM region of the two piles. Pile A (thicker one, *D* / *t*_*w*_ = 8) shows a dense region below the pile due to compaction in the ‘nose cone’ region [67]. The presence of a ‘nose cone’ suggests the pile is in a (partially) plugged state. Particles inside Pile B (thinner one, *D* / *t*_*w*_ = 20) have a similar dilatant behaviour to Pile A, though the rock beneath the pile tip remains in a loose state which would suggest an unplugged state for the Pile. The contradictory conclusions between Figs. 7 and 8 demonstrate that the IFR and PLR, which are strongly dependent on material dilatancy behaviour, have limitations in evaluating plugging states in soft rocks. It should be noted that porosity contours of regions close to the pile walls are affected by rigid wall effects [25] combined with particle upscaling, that will both tend to increase the local porosity measures.

#### Damage zone

**Fig. 9:**
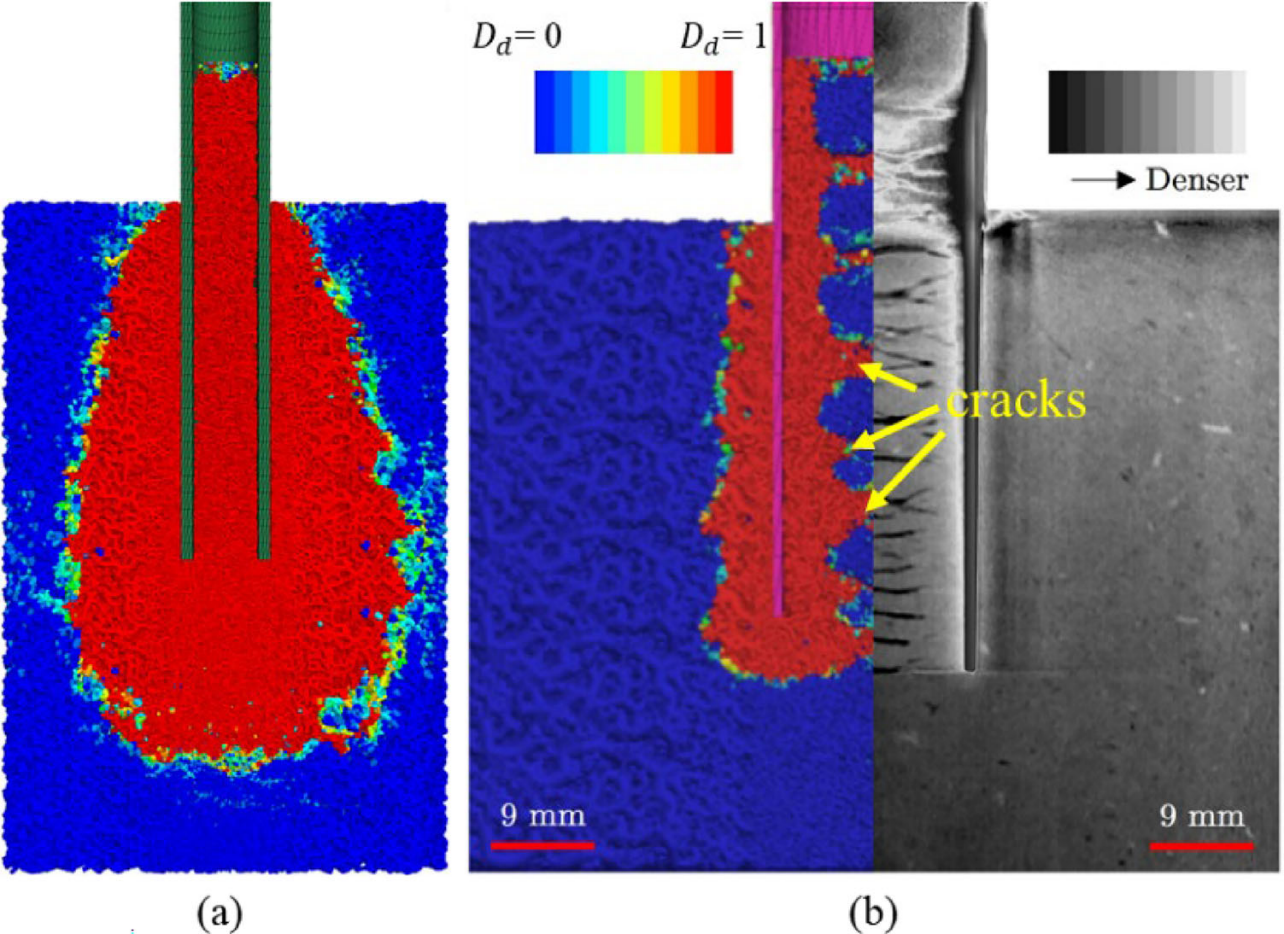
Damage zone distribution in the DEM region: **a** Pile A (*D* / *t*_*w*_ = 8), **b** Pile B (*D* / *t*_*w*_ = 20). The µCT image is taken from [56]

Figure 9 shows contours of bond damage state, ${D}_{d}$. For Pile A, the damage zone appears to form a continuous bulb of damaged rock ahead of the pile tip, consistent with the ‘nose cone’ of reduced porosity in Fig. 8a. The width of damage zone external and adjacent to the shaft appears to be related to $D/{t}_{w}$—the pile with a larger $D/{t}_{w}$ (Pile B) has a thinner damage zone beneath the pile which is focussed around the pile tip annulus, consistent with an unplugged condition.

Internal bond damage appears to be related to $D/{t}_{w}$ and plug state. Pile A with a lower $D/{t}_{w}$ has the most severe and uniform internal damage, and this damage zone expands with increasing internal shaft resistance. Traditional theory suggests that the rock below the pile wall undergoes local shear failure [37]. However, due to the small ${t}_{w}/{\mathrm{d}}_{50}$ in the current study, the high stress beneath the pile wall is more likely to be caused by pure compression failure. The left half of Fig. 9b shows the damage zone of Pile B when penetrated by 20 mm compared to CT scanning of the pile in the experimental testing [56]. The width of the internal damage zone in the experiment is approximately equal to the pile thickness, while it is larger in the simulation due to the particle upscaling. Experimentally observed internal cracks in the right half of Fig. 9b are captured in simulations, indicating the performance of this model to replicate the initialisation and propagation of cracks. These cracks are macroscopic responses to microscopic bond breakages.

The horizontal cracks that develop inside the plug may also contribute to the noisier response for Pile B that is shown in Fig. 5b, in addition to the effect of relatively lower ${t}_{w}/{\mathrm{d}}_{50}$ that was previously noted. Furthermore, particle scaling affects the thickness of these openings which are larger than those observed experimentally. This, in turn, may induce larger transient drops in penetration resistance.

#### Particle displacements

**Fig. 10:**
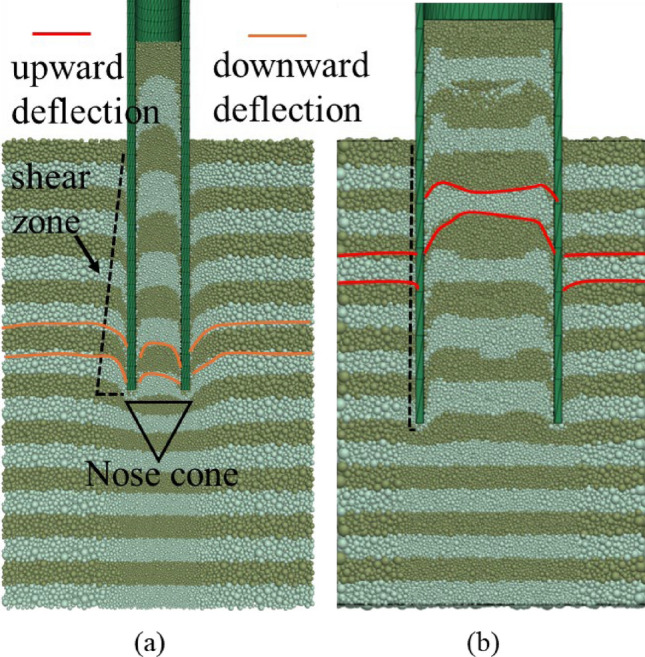
Deflection distribution in the DEM region: **a** Pile A and **b** Pile B

In Fig. 10, the position of different rock sublayers inside the pile can be explained according to the deflection effect for open-ended piles proposed by Paikowsky and Whitman [51] and Paikowsky et al. [52]. The convex upward deflections (rock moved upwards compared to its initial position) at the upper zone inside the pile are caused by the low internal shaft friction on the rising material*.* Downward deflections occur in the Pile A, indicating the relatively larger internal shaft resistance if formed inside the pile. The downward deflections (rock moved downwards compared to its initial position) form a nose cone region beneath the pile tip, corresponding to the densification zone below the annulus in Fig. 8a. Rock particles in this zone remain approximately stationary relative to the pile shaft. It also demonstrates that the presence of downward deflections is indicative of a plugged state. The distribution of deflections supports PLR and IFR being insufficient to describe the plugging state. In addition, rock particles adjacent to the external pile wall are dragged downward by shaft friction, with the shear zone width in Fig. 10 progressively decreasing upward from the pile end as indicated by the black dash line. This distribution pattern correlates with the external shaft resistance profile shown in Fig. 6b. Compared to Pile A, only downward deflections were observed within Pile B, with no discernible nose cone formation detected beneath its tip.

#### Axial load transfer mechanism

**Fig. 11:**
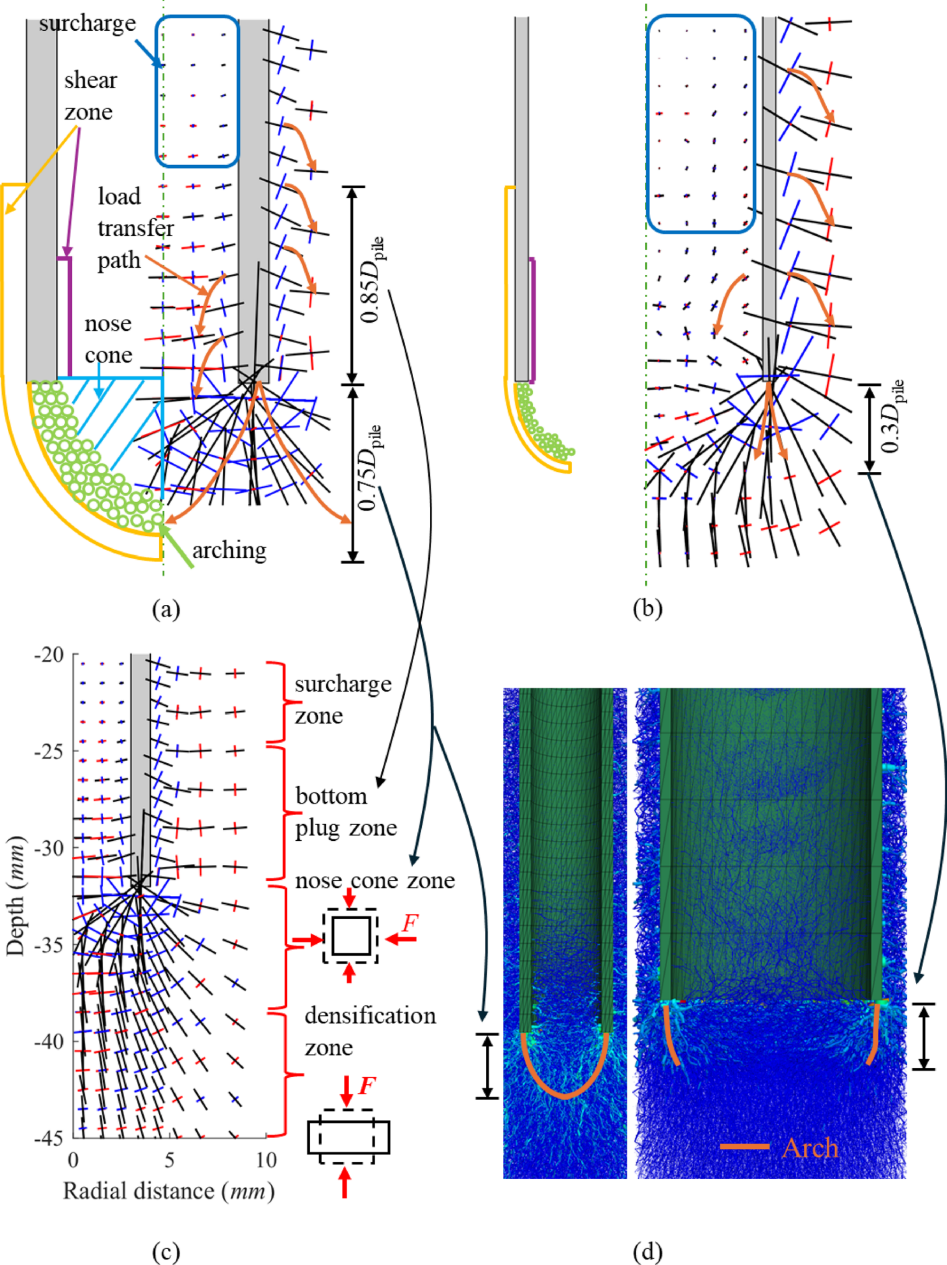
Sketch of axial load transfer mechanism: **a** Pile A; **b** Pile B; **c** category of principal stress (line length represents stress magnitude but two piles are plotted with different scales, red, blue and black colours mean the principal stress in x-, y- and z-directions); **d** force chain and arch distributions (left: Pile A; right: Pile B)

The force chain direction for load transfer closely follows the trajectory of the principal stress [52]. The magnitude and distribution of the principal stress around Pile A (thicker one, *D* / *t*_*w*_ = 8) and Pile B (thinner one, *D* / *t*_*w*_ = 20) after installation are illustrated in Fig. 11a, b. Based on the distinct distribution pattern of the principal stress, the distribution of principal stress within the range of one pile diameter can be categorised into 4 zones, as depicted in Fig. 11c (similar to Li et al. [36]). In the top zone (surcharge zone), the material experiences significant dilation (see Fig. 8a, b), resulting in smaller principal stress magnitudes and inhomogeneous rotation. This zone can be regarded as the overburden [55]. In the bottom plug zone, the vertical principal stresses near the pile wall are deflected towards the pile wall, with greater deflection closer to the pile wall, indicating the gradual development of inner friction from the pile wall inward. All three principal stresses in the nose cone are significantly deflected, indicating that the rock in this region mainly experiences a mixed type of deformation. Material in the nose cone region is subjected to radial force from the arch beneath the wall, the downward force from the plug and the lower upward reaction force combined, such that the rock in this zone undergoes combined vertical and lateral compression deformation. Finally, the rock in the densification zone is constrained by vertical compression.

Figure 11a, b presents idealised schematics of arch formation beneath the pile wall for the two piles. The arch geometry corresponds to the distribution of strong force chains beneath the wall and can be defined by the position of the outermost continuous strong force chain. Post-installation force-chain maps show that a coherent chain forms beneath Pile A, giving an arch rise of approximately 0.75*D*_pile_, whereas Pile B exhibits only localised strong chains within about 0.3*D*_pile_ from the wall (Fig. 11d). Arch stability is determined by the continuity of strong force chains: an unbroken compressive path connecting the two arch bases indicates a more stable arch; interruption by cracks/voids implies an unstable arch.

Figure 11 further reinforces the concept that both external and internal friction gradually develop from the bottom to the top and from the pile wall towards the centre of the pile. The formed complete arch below the pile prevents external particles from entering the internal pile cavity, leading to a constant plug height.

#### State of destructured rock during installation

**Fig. 12:**
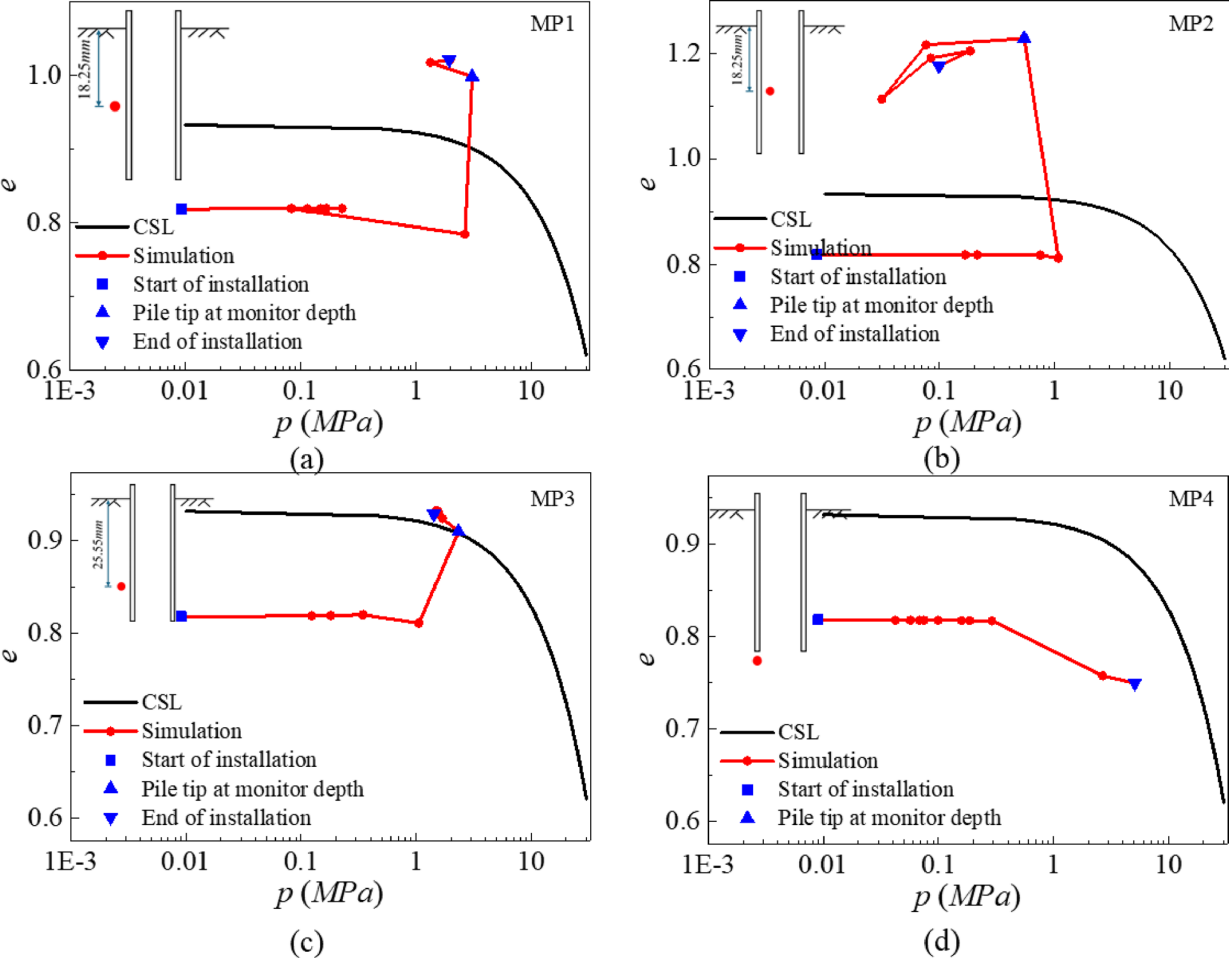
Mean stress–voids ratio space during installation: **a** MP1; **b** MP2; **c** MP3 and **d** MP4

To explore the origin of installation resistance in sands, Martinelli and Pisanò [42] and Spyridis and Lopez-Querol [58] analysed the evolution of stress paths in sand during pile penetration with reference to the CSL and evaluated the prediction of the state parameter for volumetric response. A similar analysis for Pile B is given in Fig. 12. The arrangement of monitoring points (MP) is as follows: MPs 1–3 along the pile shaft are positioned at a horizontal distance of one pile wall thickness from the pile surface, while MP4 is located vertically beneath the pile tip at a distance of one pile wall thickness. The radii of these MPs are 3d_50_. To accurately characterise the evolution of rock state, a three-stage marking method is adopted in the figure to clearly illustrate the stress–void ratio paths at each monitoring point: squares represent the initial state, upward triangles indicate the state when the pile tip reaches the depth of the monitoring point, and downward triangles correspond to the unloading stage after the pile tip passes the monitoring point.

Monitoring data show that significant dilative behaviour is observed at all monitoring points except for MP4 directly beneath the pile tip (Fig. 12d). Specifically, during the initial stage of penetration (before the pile tip reaches the depth of the monitoring points), the void ratio at each point remains relatively constant with increasing mean stress. Once the pile tip reaches the depth of a monitoring point, the void ratio exhibits a sharp increase. Among them, MP2 shows the greatest rate of increase due to crack formation within the pile induced by penetration. In the unloading stage, except for MP2 where crack presence leads to a disrupted stress path, the dilative behaviour at other points tends to stabilise. It is noteworthy that MP4 undergoes continuous densification under the high pressure from the pile tip.

The study identifies three key phenomena: first, the stress states at MPs 1–3 remain significantly higher than their initial values even after unloading; second, the pronounced dilation at MPs 1–3 is likely to trigger internal structural collapse within the soft rock, which may help explain the “free fall” sometimes reported in the field. The state parameter is defined as $\Psi =e-{e}_{cs}$, where ${e}_{cs}$ and $e$ are the critical-state and current void ratios under the same stress, respectively. $\Psi$ > 0 in the initial state indicates a looser state with a contraction tendency upon shearing, whereas $\Psi$ < 0 initially indicates a denser state with a dilation tendency. All cases show a positive change in $\Psi$ as installation proceeds, indicating dilation. It should be emphasised that, although $\Psi$ is commonly used to describe the volumetric tendency of sands [6], it does not explain the abnormal dilation tendency observed at MPs 1–2 ($\Psi$ > 0) in destructured chalk. This abnormality is attributed to crack formation during the penetration of Pile B.

## Lateral loading results

### Global lateral response

**Fig. 13:**
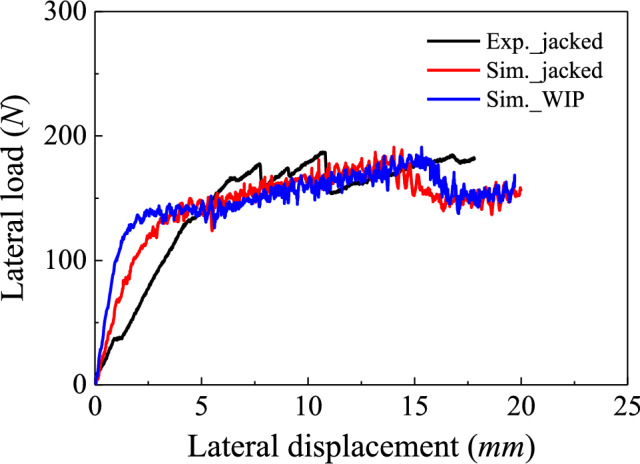
Load vs. pile top displacement

Pile lateral response following previous installation effects is investigated here by comparing the jacked Pile B (with higher $D/{t}_{w}$ closer to real OWT monopiles) and wished-in place (WIP) simulations. In Fig. 13, the WIP case has a 50% greater initial stiffness due to the intact microstructure of the surrounding rock compared to the jacked case. Installation effects appear to induce a minimal change in the ultimate lateral capacity (< 2% when the pile top displacement is 0.1${D}_{\mathrm{pile}}$), as also reported in the pile simulations in sand conducted by Staubach et al. [59]. The simulation results appear to accurately predict the experimental data in terms of the initial stiffness, yield load and ultimate load. Consistent with the microstructural observations (Fig. 9), the stiffness reduction in installed Pile A (relative to its WIP counterpart) should exceed that of Pile B, owing to more extensive plug-induced damage.

### Micromechanical results

#### Rock flow mode

**Fig. 14:**
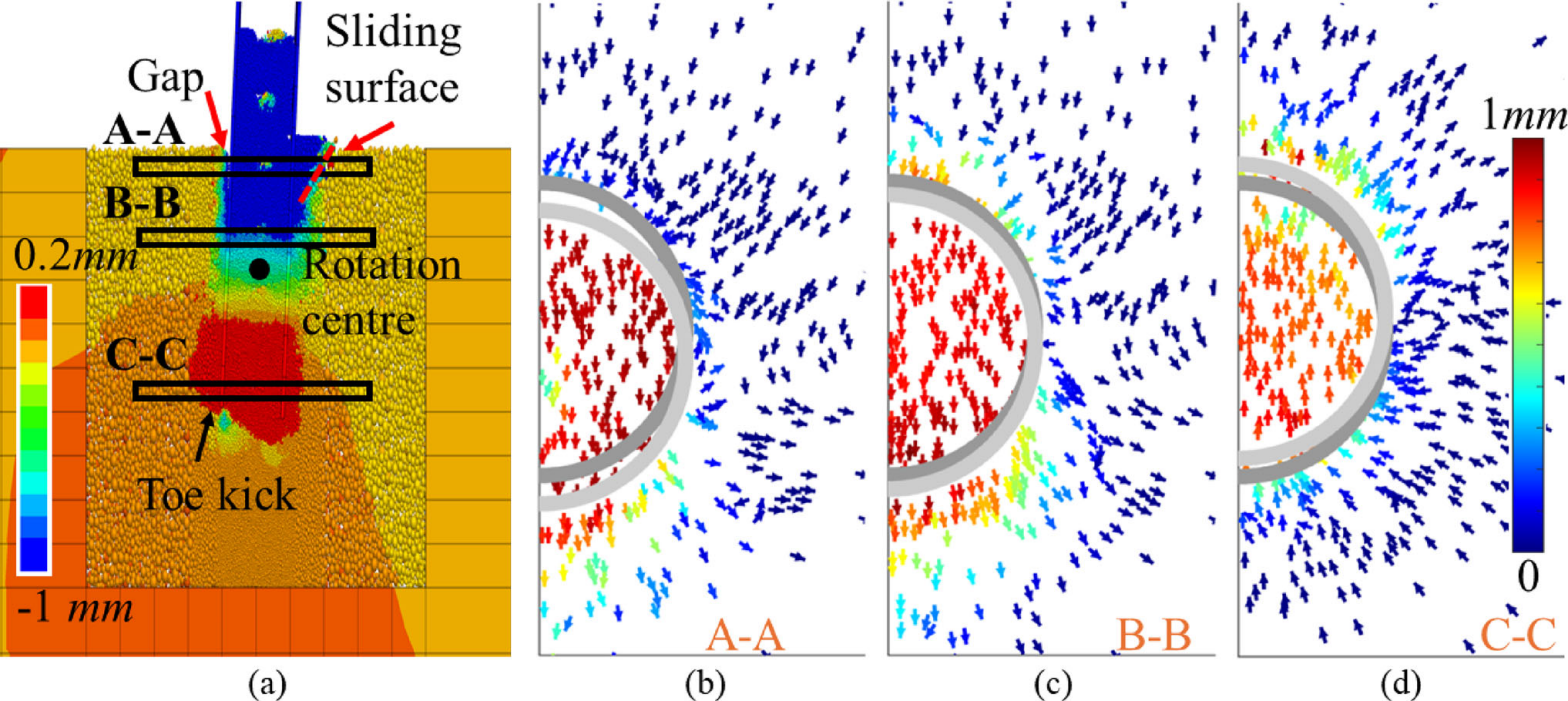
**a** side view of displacement field; **b** to **d** rock flows on different planes (dark and light grey semicircles are the original and current positions of the pile, respectively)

As presented in Fig. 14, the pile undergoes rigid-body rotation with a centre of rotation located at a depth of 0.5${L}_{p}$ below the ground surface. A gap is observed on the active side between the rock near the ground surface and the pile shaft, which is expected to significantly influence the shape of the load deformation (*p*-*y*) response during cyclic loading [15, 43]. On the passive side, the rock near the ground surface exhibits heave, allowing the slip surface to be clearly inferred. The area enclosed by this surface corresponds to the wedge-shaped failure zone of the soft rock. Figure 14b–d presents particle movement patterns across cross sections at different depths, where arrows indicate displacement directions, and arrow colours represent displacement magnitudes. The flow modes on the three planes are similar, and no flow-around behaviour—commonly observed for laterally loaded piles in soils [23]—is identified at any depth. According to Murff and Hamilton [47], this suggests that the ultimate lateral capacity should be similar at varied depths, a conclusion which is confirmed by the field tests conducted by McAdam et al. [43].

Figure 14a additionally demonstrates that the lateral displacement is primarily concentrated near the pile. According to Pedone et al. [53], when the lateral displacement at the mudline reaches 0.1*D*_pile_, the plastic deformation zone extends up to a maximum of approximately 25*t*_*w*_ beyond the pile wall. In the present simulation, the lateral displacement at the mudline is approximately 0.02* D*_pile_, and the FDM region is located 33*t*_*w*_ away from the pile wall. Furthermore, the deviatoric stress in elements near the DEM-FDM interface at the mudline is approximately 1.2 MPa, which is lower than the triaxial yield stress under corresponding low confining pressures. These observations support the appropriateness of the elastic model used in the FDM region.

#### Stress and bond damage fields

**Fig. 15:**
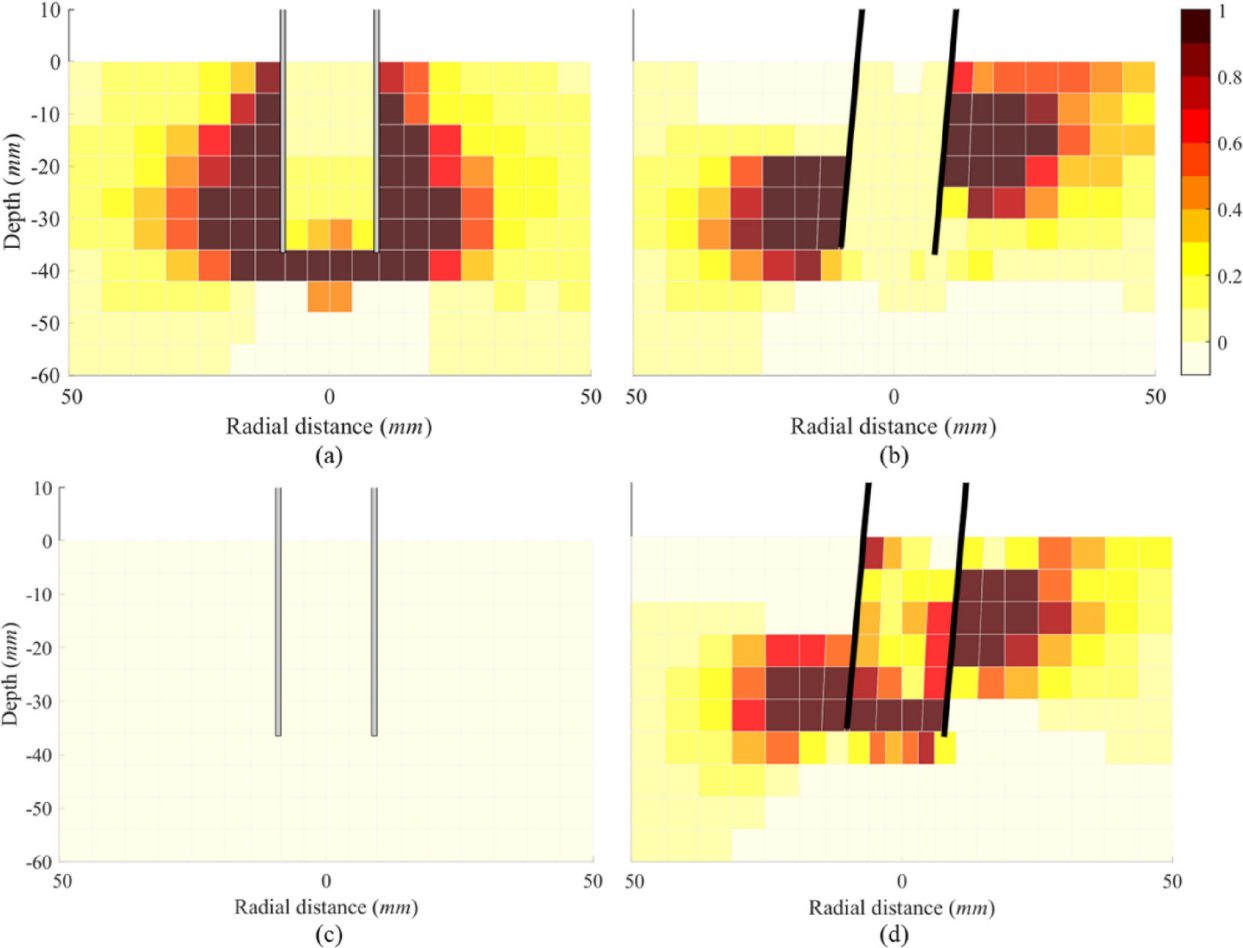
Radial stress field of the Pile B: **a** after installation; **b** after lateral loading of the jacked case; **c** after installation; **d** after lateral loading of the WIP case (unit: MPa)

**Fig. 16:**
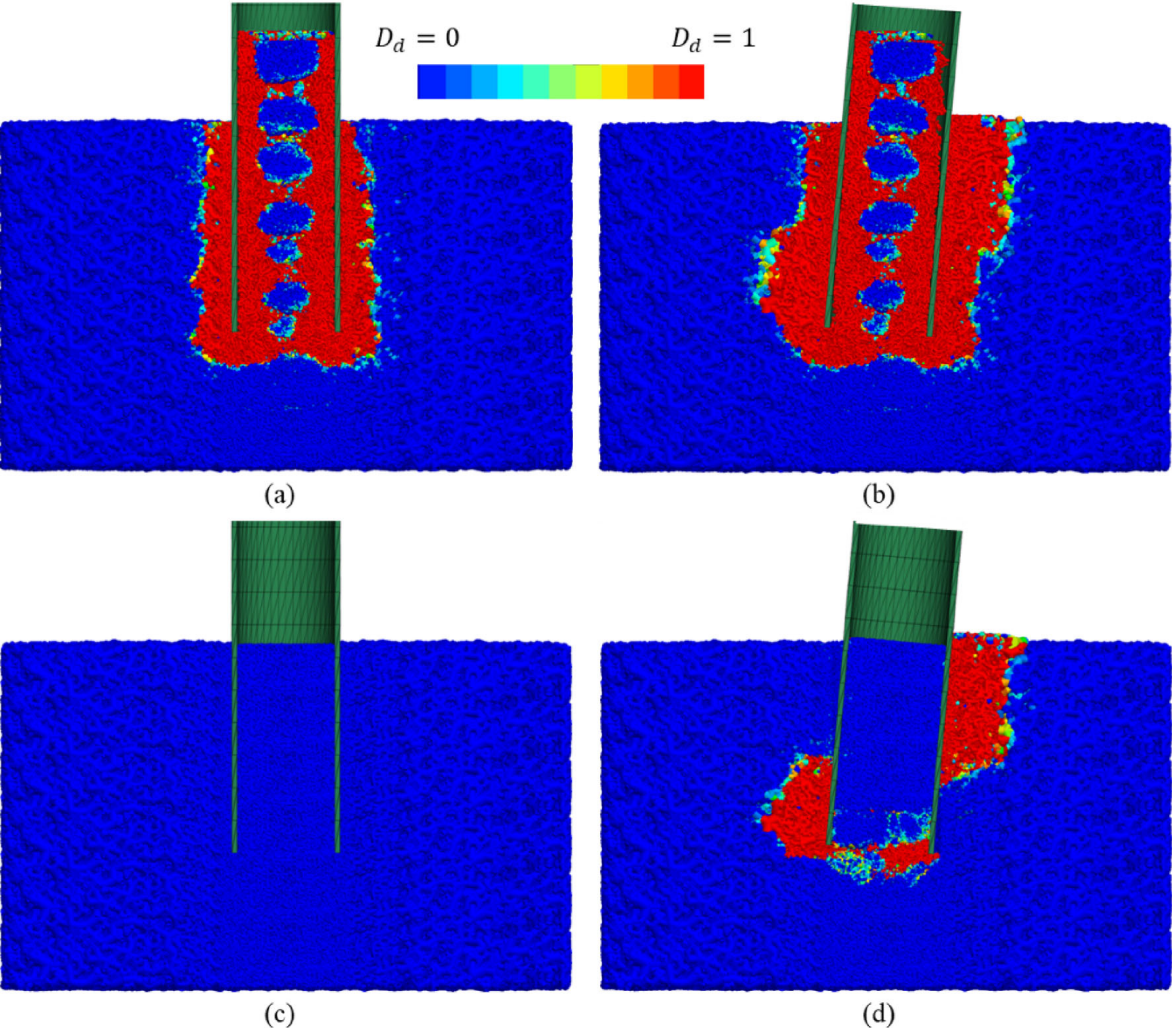
Distribution of damage zone of the Pile B: **a** after installation; **b** after lateral loading of the jacked case; **c** after installation; **d** after lateral loading of the WIP case

Comparing Fig. 15a, b, a significant radial stress increase is observed in the rock below the zero-deflection point on the passive side and above it on the active side in the jacked pile. These regions are the main areas that provide lateral resistance and demonstrate that the plug has no significant effect on lateral capacity of the jacked pile. In addition, intense shearing occurs below the pile tip due to toe-kick. Figure 15 also suggests that a pile which would be typically considered unplugged still can produce a significant alteration to the stress field during installation, which is opposite to the point proposed by Hu et al. [24]. During lateral loading, the area where stress increases significantly corresponds to the area where bond damage propagates (Fig. 16). For the WIP pile case, the distribution of external high-stress zones closely matches that of the jacked pile (Fig. 15d), with identical patterns observed in the external damage regions (Fig. 16d). However, significant stress field modification is observed within the plug, particularly near the pile tip. Comparative analysis of Fig. 15b–d conclusively demonstrates that the plug has a substantial effect on the lateral response of the WIP pile during initial loading stages (prior to rock damage beneath the plug).

#### Particle connectivity

**Fig. 17:**
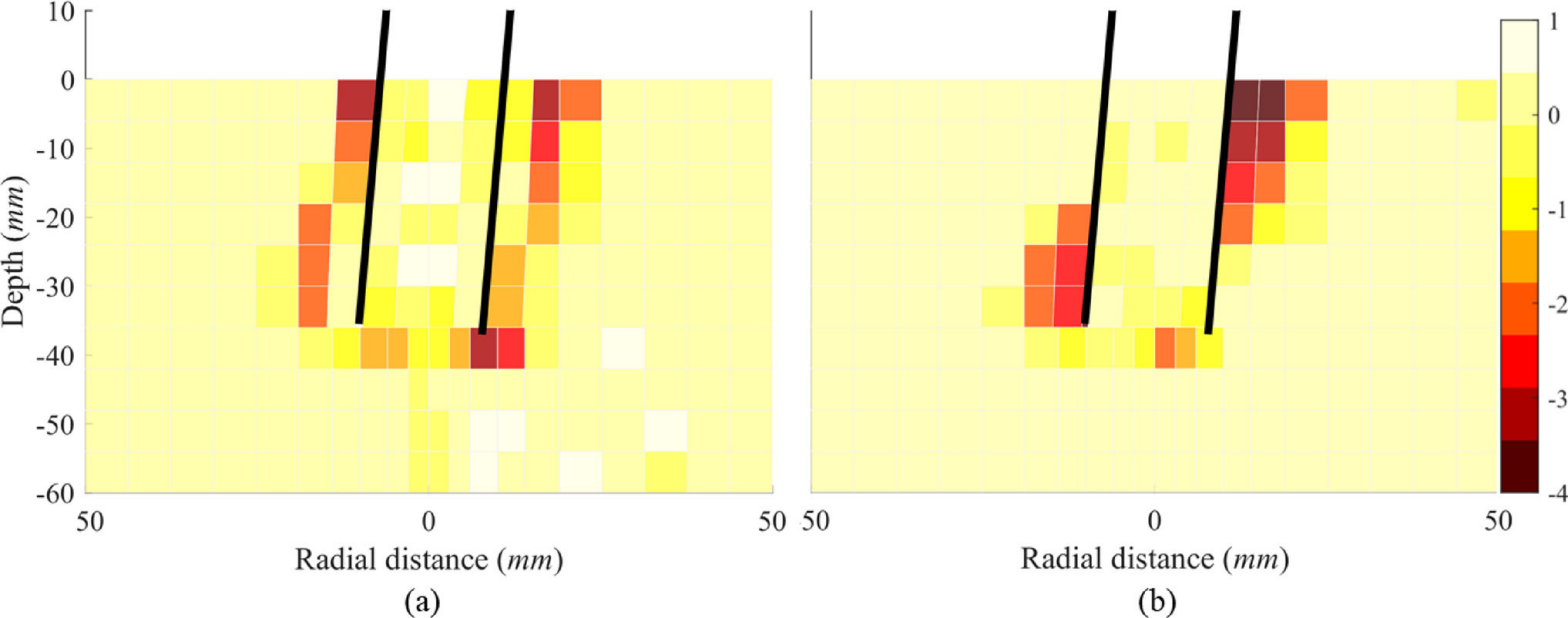
Distribution of the change in contact connectivity of **a** jacked and **b** WIP Pile B

Particle connectivity is defined as the average coordination number of all particles within each probe bin. Here, the change of particle connectivity during lateral loading is used to interpret stress changes around the pile for a jacked pile and for a WIP pile. Figure 17 shows the distribution of the change in connectivity between the end of pile installation and the end of lateral loading. The change in connectivity for each bin is calculated by subtracting the pre-lateral loading connectivity from the post-lateral connectivity, where negative values indicate contact reduction. The regions with reduced connectivity in Fig. 17b correspond closely to the areas of stress increase shown in Fig. 15d, whereas the regions experiencing stress relief in Fig. 15b align with those showing reduced connectivity in Fig. 17a. A significant drop in connectivity within the wedge-shaped failure zone and persistently stable connectivity near the pile tip are clearly observed in Fig. 17a. In these regions, the breakage of inter-particle bonds during the installation process creates a crushed rock annulus that inhibits the increase in connectivity even in the passive regions around the pile. This is likely because the putty has reached a critical state (see Fig. 12) where it is known that even connectivity reaches its “steady” state [14]. Therefore, unlike that in the WIP pile, particle connectivity in passive zones shows no increase adjacent to the jacked-pile walls during lateral loading.

**Fig. 18:**
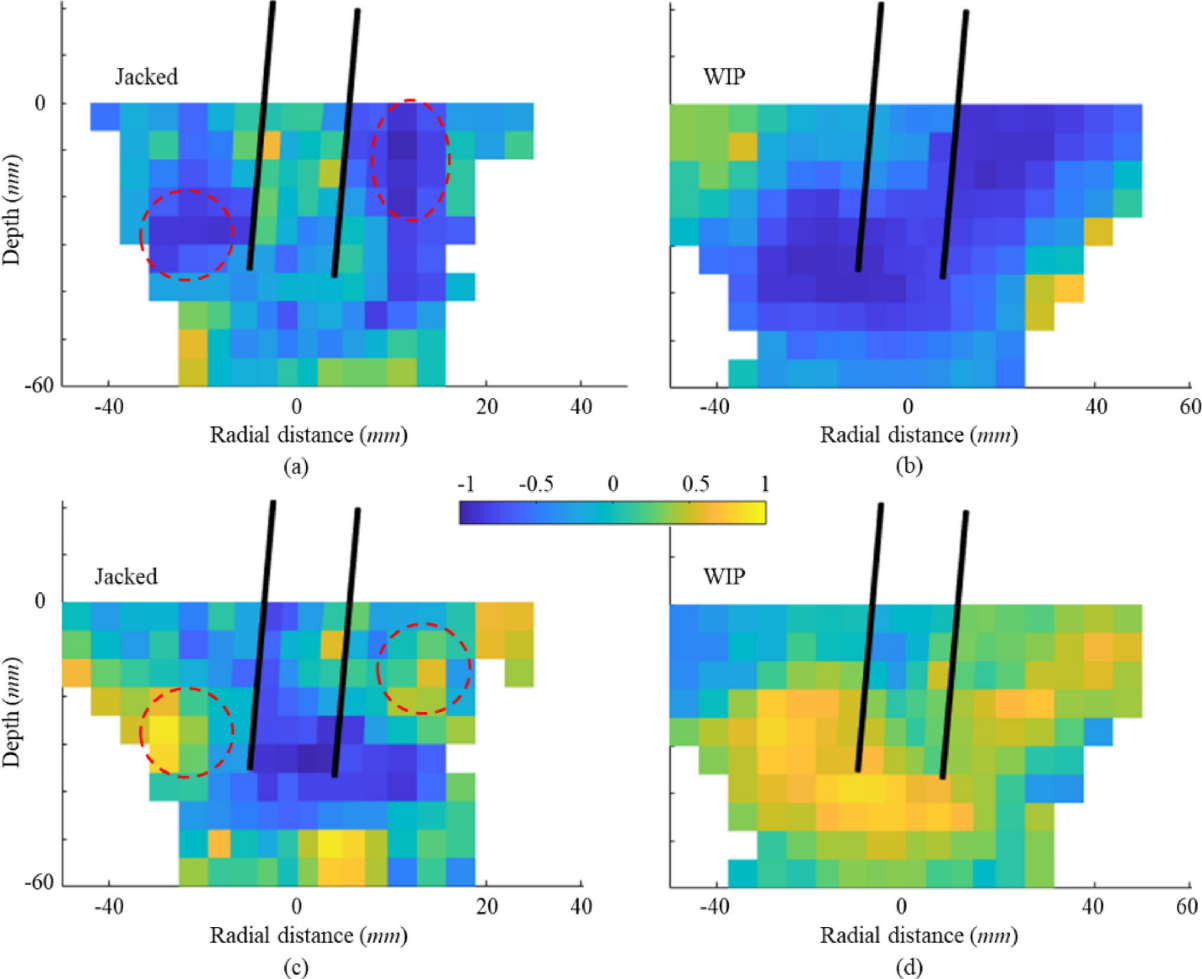
Spearman correlation coefficient distribution: **a** and **b** the change in connectivity and damage of the jacked and WIP cases; **c** and **d** the change in connectivity and stress of the jacked and WIP cases

To further explore the link between connectivity and changes in bond damage and stress, Fig. 18 presents, for each probe bin, (i) the relationship between changes in contact connectivity and damage, and (ii) the relationship between changes in connectivity and changes in stress around the pile (see the figure below) using a Spearman correlation. The Spearman correlation coefficient ranges from − 1 to 1. A value greater than 0 indicates a positive monotonic relationship, while a value less than 0 signifies a negative one. The stress change was calculated as the difference between the stress before lateral loading and the stress after lateral loading (initial stress minus final stress).

The cell-based analysis results are shown in Fig. 18, with white areas indicating not a number (NaN). In the WIP pile case, particle connectivity demonstrates significant correlations with both damage and stress variations (Fig. 18b, d): an increase in damage shows a strong negative correlation with a decrease in connectivity, while a reduction in stress (negative in all grids for this case) exhibits a strong positive correlation with connectivity loss. For the jacked pile case, the passive zone in Fig. 18a reveals a strong negative correlation between damage change and connectivity change (marked by red circle), whereas no clear correlation is observed near the pile wall, consistent with the stabilised connectivity around the pile shown in Fig. 17a. Meanwhile, the strong positive correlation between stress and connectivity (red circle in Fig. 18c) also corresponds to the stress increase zone in Fig. 15b. Moreover, the notable negative correlation below the pile tip in Fig. 18c indicates that lateral loading caused stress relief in this zone (defined as a positive stress change), transitioning the particle structure from dense to loose and thereby reducing connectivity.

## Conclusions

A 3D coupled DEM-FDM model for open-ended piles installed in soft rocks has been built based on a recently developed scalable bond damage model. The installation effects for two different model piles section geometries have been investigated, including the distribution of the stress field, porosity changes, the axial load transfer mechanism and effects on subsequent monotonic lateral loading. The numerical results are validated against two 1 g small-scale model tests, yielding the main conclusions as follows:

1. Limitations of traditional plugging indices: Due to the pronounced dilative behaviour of crushed particles in soft rocks, traditional plugging indices such as PLR and IFR fail to accurately describe the actual plugging state of OE piles. This study demonstrates that the interaction between internal arching structures and the nose cone ahead of the advancing pile base provides a more physically representative description of the plugging mechanism.
2. Microscale-based axial load-transfer mechanism: A microscale-based axial load-transfer mechanism for OE piles in soft rocks is proposed. The nose cone beneath the pile tip contributes to end bearing capacity (for plugged piles only), while external shearing provides external shaft resistance (for both plugged and unplugged piles) with negligible contribution from internal shaft friction. The magnitudes of both components are influenced by crack formation during installation. As most OWT piles have large values of *D*/*t*_w_ and therefore will not reach a fully plugged (or even partly plugged state), this study also microscopically validates the rationale behind the current ICP-18 design method for chalk, which assumes negligible internal shaft resistance.
3. Impact of installation effects on lateral stiffness: Although installation effects have limited influence on the ultimate lateral capacity, they significantly reduce the initial lateral stiffness by inducing microstructural damage and stress redistribution. This has important implications for predicting rock deformation around the pile under cyclic lateral loading and for assessing dynamic characteristics of OWT structures.
4. Micro–Macro-response linkage: By correlating changes in contact connectivity and bond damage distribution with local stress redistribution, failure zone expansion, and lateral stiffness reduction, this study establishes a clear link between microscale damage processes and the pile’s macroscopic mechanical behaviour. The contrasting evolution of contact connectivity in corresponding zones around jacked and WIP piles demonstrates that installation-induced fine-grained annulus formation constitutes the primary factor governing their distinct initial lateral responses.

Particle upscaling improved computational efficiency, but it also reduced the pile wall-to-particle size ratio and may introduce non-physical artefacts such as overly extensive bond-damage zones and unrealistically high porosity near the pile wall. Therefore, particle upscaling has inherent limits, and an appropriate balance between efficiency and accuracy must be carefully maintained in applying the approach presented herein to boundary value problems.

## Data Availability

The data that support the findings of this study are available from the corresponding author upon reasonable request.

## List of symbols

$C$: Cohesion of the bond
${\mathrm{d}}_{00},{\mathrm{d}}_{50}$: Minimum and mean particle size
$${D}_{\mathrm{pile}}$$: Pile diameter
$$e$$: Void ratio
${E}_{\mathrm{mod}}, {\overline{E} }_{\mathrm{mod}}$: Effective moduli of unbonded contacts (inter-particle or particle–pile contacts) and bonded contacts, respectively
${f}_{L}$: Axial compression loading factor
${g}_{a}$: Activation gap of the far-field interaction
$${I}_{\mathrm{iner}}$$: Inertial number
${L}_{p}, {L}_{\mathrm{pile}}$: Installed length and pile length
$p, q$: Mean and deviatoric stresses
${R}_{o},{ R}_{i}, {R}^{*}$: External pile radius; internal pile radius; equivalent pile radius
${t}_{w}$: Pile wall thickness
${u}_{c}^{n}, {u}_{c}^{s}$: Softening rate-related parameters along normal and shear directions in the proposed contact model
${v}_{\mathrm{pile}}$: Pile jacking velocity
$${\varepsilon }_{a}$$: Axial strain
${\theta }_{c}^{b}$: Softening rate-related parameters along rotation direction in the proposed contact model
${\kappa }^{*}, {\overline{\kappa }}^{*}$: Normal-to-shear stiffnesses of unbonded contacts (inter-particle or particle–pile contacts) and bonded contacts, respectively
$\mu , {\mu }_{\mathrm{pile}-\mathrm{chalk}}$: Friction coefficients between the particles and the pile–chalk interface
${\sigma }_{c}, {\sigma }_{t}$: Compressive and tensile strengths of the bond
${\sigma }_{r}, {\sigma }_{3}$: Radial stress acting on the pile and confinement
${\tau }_{z}$: Shear stress acting on the pile
$$\rho$$: Sample density
$$\Psi$$: State parameter
